# Plasticization of Polylactide after Solidification: An Effectiveness and Utilization for Correct Interpretation of Thermal Properties

**DOI:** 10.3390/polym12030561

**Published:** 2020-03-04

**Authors:** Marta Safandowska, Artur Rozanski, Andrzej Galeski

**Affiliations:** Centre of Molecular and Macromolecular Studies, Polish Academy of Sciences, Sienkiewicza 112, 90-363 Lodz, Poland; andgal@cbmm.lodz.pl

**Keywords:** polylactide, plasticization, thermal properties

## Abstract

Polylactide/triethyl citrate (PLA/TEC) systems were prepared in two ways by introducing TEC to solidified polymer matrix (SS) and by blending in a molten state (MS) to investigate the effectiveness of the plasticization process after solidification of polylactide. The plasticization processes, independent of the way of introducing the TEC into PLA matrix, leads to systems characterized by similar stability, morphology, and properties. Some differences in mechanical properties between MS and SS systems result primarily from the difference in the degree of crystallinity/crystal thickness of the PLA matrix itself. Based on the presented results, it was concluded that the plasticization process after solidification of polylactide is an alternative to the conventional method of modification-blending in a molten state. Then, this new approach to plasticization process was utilized for the interpretation of thermal properties of PLA and PLA/TEC systems. It turned out that double melting peak observed at differential scanning calorimetry (DSC) thermograms does not result from the melting of a double population of crystals with different lamellar thickness, or the melting of both the α′ and α crystalline phase (commonly used explanations in literature), but is associated with the improvement of perfection of crystalline structure of PLA during heating process.

## 1. Introduction

Petroleum-based polymers, such as polyethylene (PE), polypropylene (PP), polystyrene (PS), polyethylene terephthalate (PET) etc., are used in almost all fields in daily life. The production of these polymers is greater than 100 billion pounds per year, and it is expected that this number will keep rising with the development of polymer industry. Considering the environmental pollution caused by non-degradable petroleum-based polymers, biodegradable and renewably derived polymers are currently being sought for. One such polymer is polylactic acid (PLA), a bio-based and biodegradable polyester, primarily produced by industrial polycondensation of lactic acid and/or ring-opening polymerization of lactide [1,2,3]. PLA because of its properties (biocompatibility, thermoplastic fabricability, high strength, good crease-retention) can be considered for potential applications in fields of medical, packaging, agriculture products, textiles, advanced energy materials, as well as automotive and aerospace. However, the inherent brittleness, slow degradation rate, hydrophobicity and lack of reactive side-chain groups hinders its wide application possibilities [4]. To enhance the flexibility and toughness in PLA several procedures, including copolymerization, polymer compositing, and polymer blending are used. Blending is an easy and cost-effective method of fabricating materials, and therefore it is the most commonly chosen method of modifying PLA properties [5,6]. In the literature, many substances have been used for blending with PLA, and among them polymers (biodegradable and non-biodegradable) and plasticizers can be distinguished. The plasticizer should be compatible with the polymer matrix. The compatibility of these two components in the blend has an impact on the physical properties of the polymer (glass transition temperature of the amorphous phase (*T*_g_), melting point of the crystalline region (*T*_m_), crystallinity), which consequently determine the material’s properties such as processability, rigidity, tensile strength, etc. The molecular weight, polarity, and end groups of plasticizer significantly affects the mechanical properties of the plasticized polymer and is therefore of great importance from the point of its potential applications. Low molecular weight compounds, such as lactic acid, glycerol [5], polyethylene glycol (PEG) [7,8], triacetine (TAc) [9], tributyl citrate (TbC) [10,11], triethyl citrate (TEC) [12,13], and diethyl bishydroxymethyl malonate (DBM) [14] have been investigated as potential plasticizers for PLA. It was demonstrated that these compounds reduce *T*_g_, *T*_m_, and crystallization temperature (*T*_c_) of PLA-based materials and as a consequence, contribute to the increase of its ductility and improve processability. Unfortunately, the studied blends were not stable over time because the plasticizer tends to migrate to the material surface. Ljunberg et al. [11,14] showed that PLA-based materials undergo cold crystallization because of the increased chain mobility, and the decreasing volume of the amorphous phase compels the plasticizer to migrate. The authors revealed that the migration can be reduced by increasing the molecular weight of the plasticizers, i.e., the use of oligomeric plasticizers such as TbC-oligoesters, DBM-oligoesters, or oligomers of lactic acid (OLAs) [15,16]. However, the reduction in solubility because of the increased weight can lead to phase separation and formation of a two-phase system [16,17,18]. It seems that the migration of low molecular weight compounds from the polylactide matrix can be limited through controlled crystallization [14]. It is known that in order to stabilize the polymer material it is important to control its crystallinity grade. The ability of PLA to crystallize depends on its enantiomeric composition (_L_-lactide, _D_-lactide) and decreases with increasing content of repeating units of different chirality [19]. The cold crystallization method causes intense nucleation of spherulites, and as a result leads to the formation of stable crystalline structure [20].

The goal of this study is plasticization of cold-crystallized polylactide. As already mentioned, the plasticization of PLA in order to achieve suitable properties (processability, ductility, rigidity, tensile strength, etc.,) for different applications is most often carried out by using the polymer blending technique. In previous papers [21,22,23,24] we presented a new way of modification of amorphous regions by infusing low molecular weight modifier into solidified polymer matrix. The influence of physical state of the amorphous phase on thermo-mechanical properties, free volume, and cavitation phenomenon in systems including polypropylene, polyethylene, and polyamide 6 with various penetrants was explained. It seems that the process of polylactide plasticization involving the introduction of plasticizer into the polymer matrix after the formation of the crystalline structure, could be an alternative to conventional methods of modification—blending in a molten state. In this work, polylactide/triethyl citrate (PLA/TEC) systems were prepared (by infusing TEC to solidified polymer matrix or by blending before the solidification) to investigate the effectiveness of the plasticization process after solidification of polylactide. Then, this new approach to plasticization process was utilized for correct interpretation of thermal properties of PLA and PLA/TEC systems.

## 2. Materials and Methods

### 2.1. Materials

Polylactide 4032D (PLA), with melt flow index (MFI) of 19.3 g 10 min^−1^ (210 °C, 2.16), weight average molar mass (*M*_w_) of 105 kg mol^−1^, and dispersity (*M*_w_ *M*_n_^−1^) of 1.35 according to size exclusion chromatography (SEC) with a multi-angle laser light scattering (MALLS) detector in methylene chloride, produced by Nature-Works LLC (Minnetonka, MN) was used. D-lactide and residual monomer contents were 1.4% and 0.14%, respectively. Triethyl citrate, (TEC), purity ≥98% was purchased from Merck KGaA and used as liquid low molecular weight modifier of polylactide.

### 2.2. Sample Preparation

The modification was carried out using two different ways of incorporating triethyl citrate into the polylactide matrix:

Modification in solid state (PLA_SS). The process of modification was performed by immersion of polylactide films (obtained by compression molding, cooled between metal plates, and subjected to a cold crystallization process) in a modifier bath placed in an oven at 70 °C for 34 days in order to obtain full penetration of the amorphous phase of the material with a low molecular weight plasticizer–triethyl citrate. The content of modifier has been estimated based on samples weight increase after completion of the modification process. The reference samples had the same thermal history.

Modification in the molten state (PLA_MS). The process of modification was performed by melt blending of all components (PLA and TEC) using Brabender batch mixer (Duisburg, Germany) operating at 180 °C for 5 min at 60 rpm in nitrogen atmosphere. Then, films were prepared from the PLA/TEC composition and cold crystallized. The content of modifier has been verified by methanol extraction at room temperature and thermogravimetric measurements (TGA). Neat PLA was also processed under the same conditions to obtain a reference material.

The samples for all measurements were cut out from 1 mm thick polymer films. The films from PLA granules mixed first in a Brabender batch mixer were prepared by compression molding (90 MPa) for 3 min in a hydraulic press at 180 °C, followed by quenching between metal blocks. Before mixing PLA granules were vacuum-dried for 4 h at 100 °C. To obtain crystalline materials, the quenched films were cold crystallized by heating between aluminum blocks at T = 120 °C for 20 min, and subsequently cooling to room temperature. The all materials and prepared films were stored in dry atmosphere (relative humidity of 6–7%) in desiccators at room temperature.

### 2.3. Characterization

Thermogravimetry (TGA): Thermogravimetric analysis was performed for estimating the content of modifiers on a Hi-Res TGA 2950 analyzer (TA Instruments, New Castle, USA). Samples of approximately 20 mg were heated at 20 °C min^−1^ from room temperature to 600 °C in an air atmosphere.

Scanning electron microscopy (SEM): The morphology of neat ad plasticized PLA samples was examined with a SEM microscope (Jeol JSM 5500LV, Tokyo, Japan) operating in the high vacuum mode and accelerating voltage of 10 kV. The samples before examination were cryo-fractured, and then coated with a thin layer of gold (approx. 20 nm) by ion-sputtering (Jeol JFC-1200, Tokyo, Japan).

Polarized optical microscopy (POM): The growth rate of spherulites was measured during isothermal crystallization (120 °C) of the thin films on a Linkam CSS-450 (Waterfield, Tadworth, UK) hot stage. The films were melt annealed at 180 °C for 3 min and then cooled down to the crystallization temperature of 120 °C—this procedure leads to much less intense nucleation than that during cold crystallization, and thus enables determination of the growth rate of the spherulite. The stage was mounted in a polarizing microscope Nikon Eclipse 80i equipped with a Nikon DS-Fi1 video camera for monitoring the crystallization.

Kinetics of plasticizer desorption: The measurements of desorption of the plasticizer from the polymer matrix were carried out by gravimetric method on the basis of changes in weight of samples over time. The weight loss of samples was tested during 60 days under laboratory conditions at room temperature.

Mechanical testing: Tensile tests were carried out at 70 °C in the temperature chamber with circulating air and at a crosshead speed of 5 mm min^−1^ on a tensile testing machine (Instron model 5582, High Wycombe, UK). The shape of tensile samples was according to ISO 527-2 standard, with 1 mm thickness and 5 mm width, the gauge length was 25 mm.

Dynamic mechanical thermal analysis (DMTA): DMTA was performed on rectangular specimens cut from 1 mm thick films, in a single cantilever bending mode using DMTA TA Q-800 apparatus (TA Instruments, New Castle, DE, USA). The measurements were carried out at the frequency of 1 Hz and at the heating rate of 2 °C min^−1^ in the temperature range from −100 to 150 °C, under a fixed deformation of 0.02%.

Differential scanning calorimetry (DSC): Thermal analysis of PLA samples was conducted by using a DSC TA Q20 analyzer (TA Instruments, New Castle, DE, USA). The instrument was calibrated with temperature and heat flow using indium as standard. The samples (5–6 mg) were weighted accurately into aluminum pans and pressed slightly to ensure good contact with the DSC cell surface. The data were recorded during heating at a constant rate of 10 °C min^−1^ under nitrogen flow with an empty pan as the reference probe. The degree of crystallinity was determined according to formula: *X_c_* = Δ*H_m_/ΔH_m_*^0^ × 100, where Δ*H_m_* is the heat of fusion of the sample, Δ*H_m_*^0^ is the heat of fusion of polymer crystal. The value for Δ*H_m_*^0^ was 143 J g^−1^ [25]. DSC measurements were also performed at two different heating rates: 2 °C min^−1^ and 5 °C min^−1^.

Fourier-transform infrared spectroscopy (FTIR): FTIR spectra were collected at room temperature on a Nicolet 6700 spectrometer (Thermo Scientific, Waltham, MA, USA) equipped with a deuterated triglycine sulfate (DGTS) detector. The technique of attenuated total refraction (ATR) was used for measurements. The spectra were obtained by adding 64 scans at a resolution of 2 cm^−1^.

Wide-angle X-ray scattering (WAXS): Analysis of the crystalline structure of the PLA samples were performed using wide-angle X-ray scattering measurements by means of a computer-controlled goniometer coupled to a sealed-tube source of CuK_α_ radiation (λ = 0.154 nm), operating at 30 kV and 50 mA (Panalytical B.V., Almelo, The Netherlands). The CuK_α_ line was filtered using electronic filtering and the usual thin Ni filter.

The diffraction from crystallographic planes (200)/(110) and (203) of PLA was analyzed with the aim of determination of crystallite length in the normal directions to this planes, calculated according to Scherrer formula:

*L_hkl_* = 0.9 × *λ*/(*β* × *cos*Θ) (1)

where *L_hkl_* is a crystallite length in the direction perpendicular to (*hkl*) plane, *λ* is X-ray wavelength, *β* is the half-width of a diffraction peak, and Θ is the Braggs diffraction angle. To determine the half-width of an analyzed peaks the deconvolution process of collected profiles were performed using WAXSFit software [26,27]. The software allows to approximate the shape of the peaks with a linear combination of Gauss and Cauchy functions and adjusts their settings and magnitudes to the experimental curve with a “genetic” minimizing algorithm. The half-widths of a diffraction peaks were corrected for apparatus broadening.

Small-angle X-ray scattering (SAXS): The lamellar structure of samples was probed with 2-dimensional small angle X-ray scattering. The Kiessig-type camera with sample detector distance of 1.2 m was coupled to an X-ray CuK a low divergence microsource, operating at 50 kV and 1 mA (GeniX Cu-LD by Xenocs SA, Grenoble, France). The scattering produced by the sample was recorded with the Pilatus 100 K solid-state area detector of the resolution of 172 × 172 μm^2^ (Pilatus 100K; Dectris, Baden-Daetwill, Switzerland). Background and Lorentz corrections were applied to the curves. The long period and thickness of lamellar crystals was determined with the use of correlation function method. All details of this calculation procedure were described elsewhere [28].

Extraction process: The plasticizer from the polylactide/triethyl citrate systems was removed by extraction in a bath that contain 99.8% (GC) solution of methanol. The extraction process was carried out at room temperature for 4 days, then the samples were dried and subjected to further studies.

## 3. Results and Discussion

Triethyl citrate (TEC) was selected to modify the physical properties of the polylactide (thermal and mechanical). TEC has solubility parameters δ = 19.8 (J cm^−3^)^1/2^ close to that of polylactide δ = 20.1 (J cm^−3^)^1/2^, therefore it can easily penetrate into the amorphous phase of the polymer [10]. The modification was carried by infusing TEC to solidified polylactide matrix or by blending before its solidification. The efficiency of plasticizer penetration and its amount introduced into the matrix was estimated based on the weight changes of the PLA films recorded during the modification process. After 34 days, the polylactide matrix was completely saturated with plasticizer (Figure S1 of Supplementary Material). In the obtained polylactide/plasticizer system (PLA/TEC_SS), triethyl citrate constitutes 14.1 wt.%.

In the second method (for comparative purposes), TEC was incorporated into the polylactide in the classical way by melt blending (modification in the molten state, MS). TEC was added to the molten PLA matrix in an amount similar to that obtained for SS samples. The content of TEC in the PLA/TEC_MS samples was determined on the basis of TGA measurements (Figure S2 of Supplementary Material) as well as the change in weight of the samples recorded during extraction of the plasticizer with use of methanol as a solvent. The obtained data are shown in Table 1.

**Figure 1:**
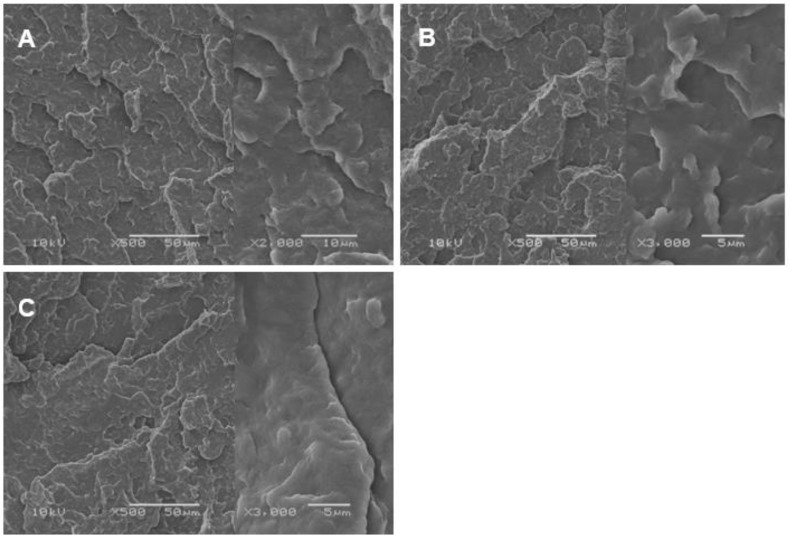
Scanning electron microscopy (SEM) micrographs of fractured surface of neat PLA (**A**) and PLA/TEC systems prepared by the modification in the solid (**B**) and molten (**C**) state.

It is known that the key factor in the selection of the plasticizing agent is its potential miscibility with the polymer matrix, and above all the stability of the plasticized system. The films prepared from pure PLA, as well as those after the plasticization process have an iridescent white color. The miscibility of the polylactide matrix with triethyl citrate was verified using a SEM microscopy. Figure 1 shows SEM micrographs of cryo-fractured surfaces of neat and plasticized PLA (solid and molten state) films.

**Figure 2:**
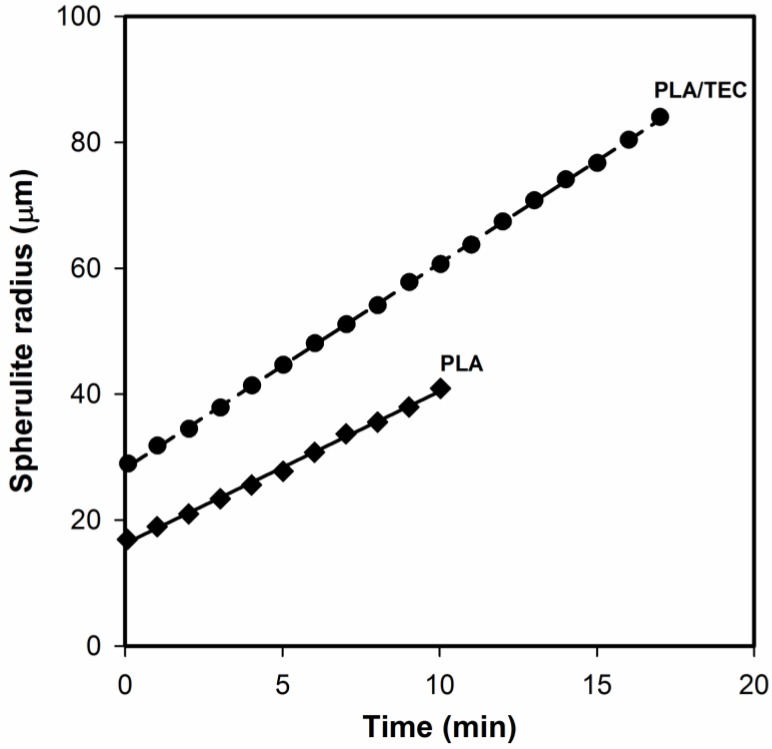
Changes in spherulite radius as functions of time for neat and plasticized PLA.

It can be seen that the surface of both PLA and PLA/TEC systems is fairly smooth without noticeable heterogeneities, which suggests that the fraction of triethyl citrate is molecularly dispersed in the polylactide matrix. The spherulite growth rate measurements confirmed the homogeneous dispersion of the plasticizer in samples of PLA/TEC. Based on the changes in spherulite radius as functions of time (Figure 2), it is clearly seen that the radius increases linearly with time also for the sample containing the triethyl citrate. This excludes, therefore, the possibility of the plasticizer accumulating in front of growing spherulite, which in consequence would lead to its segregation during crystallization.

The high miscibility of TEC with PLA results primarily from the polar interaction between the ester groups of polylactide and the plasticizer [29]. The miscibility of PLA with plasticizers also depends on the molecular weight (*M*), namely the low molecular size of the plasticizer allows it to occupy intermolecular spaces between polymer chains. Pluta et al. [30] based on SEM micrographs of PLA blends with block copolymers of ethylene glycol and propylene glycol confirmed better miscibility of PLA with copolymers having lower *M*. SEM analysis allowed to investigate the influence of the chemical structure of copolymers, which are liquid at room temperature, on the structure of the polymer materials. The authors showed that because of the liquid state of the copolymers at room temperature, the holes, rather than copolymer inclusions, in which the copolymers have accumulated, can be easily seen on SEM micrographs of samples with phase-separated modifier.

The stability of the polylactide/plasticizer systems was determined based on tracking the desorption of a plasticizer from the polymer matrix by the gravimetric method. Analysis of the desorption kinetics of triethyl citrate (Figure S3 of Supplementary Material) clearly indicates that the polylactide/triethyl citrate systems prepared by infusing triethyl citrate to solidified polylactide matrix or by blending before the solidification are characterized by similar, good stability.

**Figure 3:**
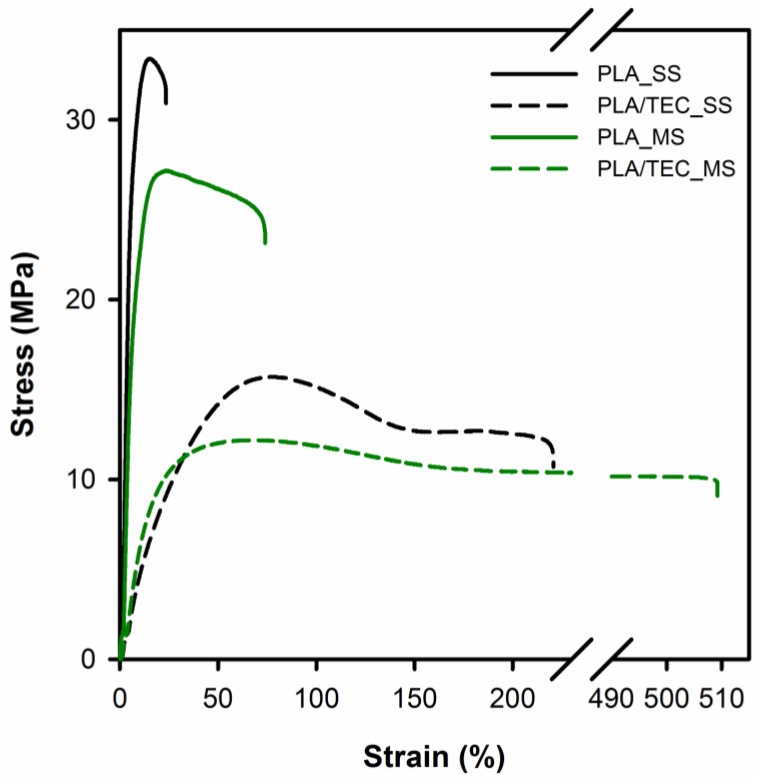
Tensile stress–strain curves for samples of unmodified polylactide and polylactide/triethyl citrate systems.

Using tensile testing machine mechanical response of polylactide/plasticizer systems in relation to the reference (neat) material was determined and the influence of the different ways of introducing the plasticizer into polylactide matrix on yield stress (*σ*_y_), stress and strain (*σ*_f_, *ɛ*_f_) at fracture was described. Figure 3 shows the representative tensile stress vs. strain curves for neat PLA and PLA/TEC systems films, the average values of yield stress, stress and elongation at fracture are summarized in Table 2.

The analysis of the tensile stress–strain curves and the data in Table 2 shows that there is a large difference between the plasticized and neat polylactide samples. It is known that a low molecular weight plasticizer behaves like a solvent when mixed with a polymer and leads to a reduction in the macromolecular chain cohesion. The plasticizer penetrates between the macromolecular chains of the polymer and causes a decrease in the cumulative intermolecular forces along them [31,32]. Accordingly, the incorporation of a plasticizer into the polymer matrix, irrespective of its solidification state, results in a reduction in stress due to the lower molecular adhesion. The data reported in the Table 2 show that plasticization leads to a two-fold decrease in yield stress from 33.4 to 16.0 MPa for solid state samples and from 27.8 to 12.1 MPa for molten state samples. Interestingly, the strain at fracture increased from 23.2 to 216.2% and from 75.2 to 488.5% for solid state and molten state samples, respectively. As stated in the papers [33,34,35,36], the decrease in yield strength observed for a particular polymer is primarily associated with a reduction in the thickness of crystals or the degree of crystallinity of these samples. In our recent works [23,37] we analyzed the effect of introducing molecules of wax or nanodecane into the amorphous phase regions of polypropylene. We revealed that low molecular weight compounds do not lead to reduction of the thickness of polypropylene crystals and degree of crystallinity. The decrease of the yield stresses in the case of samples modified in the solid state was caused by the stress generated as the result of swelling of disordered regions (amorphous phase). As a result of the increase in the interlamellar distance, the stress state of the transmitters connecting adjacent crystals (molecular network of the amorphous phase) changed, which caused a proportional change in the stress state of the crystalline component. The reduction in the yield stress observed for polypropylene/modifier blends prepared by blending in the molten state resulted, however, from the stress generated inside the amorphous phase during the solidification process in the presence of a modifier. By pushing the modifier molecules out of the growing crystals, the physical state of the amorphous phase changed, and the fragments of macromolecules that connect adjacent crystals created a strained network. In this context, the stress needed to activate the plastic deformation mechanism of the crystals is reduced by the stress exerted on the crystals by the swollen amorphous phase. This explanation seems to be appropriate also for polylactide/plasticizer systems and will be further referred to the crystal thickness results and the degree of crystallinity of these systems.

From the tensile stress vs. strain curves (Figure 3) it is noticeable that conditions of the solid state modification (heat treatment at 70 °C) influence the mechanical properties of PLA films. As shown in Table 2 the value of yield stress increases from 27.8 MPa for PLA_MS to 33.4 MPa for the PLA_SS sample, and the value of strain at fracture decreases from 75.2% for PLA_MS to 23.2% for PLA_SS. An analogous correlation in mechanical properties can be observed for PLA/TEC systems and this is probably due to the difference in the degree of crystallinity/crystal thickness of the PLA matrix itself (see DSC data, Table 3). A slightly higher crystallinity for the samples prepared by the method of solid state modification results from the phenomenon of further crystallization (annealing) of samples under the conditions of the modification process (T = 70 °C, t = 34 days).

Dynamic thermo-mechanical analysis was used to assess the impact of a plasticizer introduced in two different ways into polymer matrix (modification in solid and molten state) on polylactide viscoelastic properties and chain motion at the glass/rubbery state.

**Figure 4:**
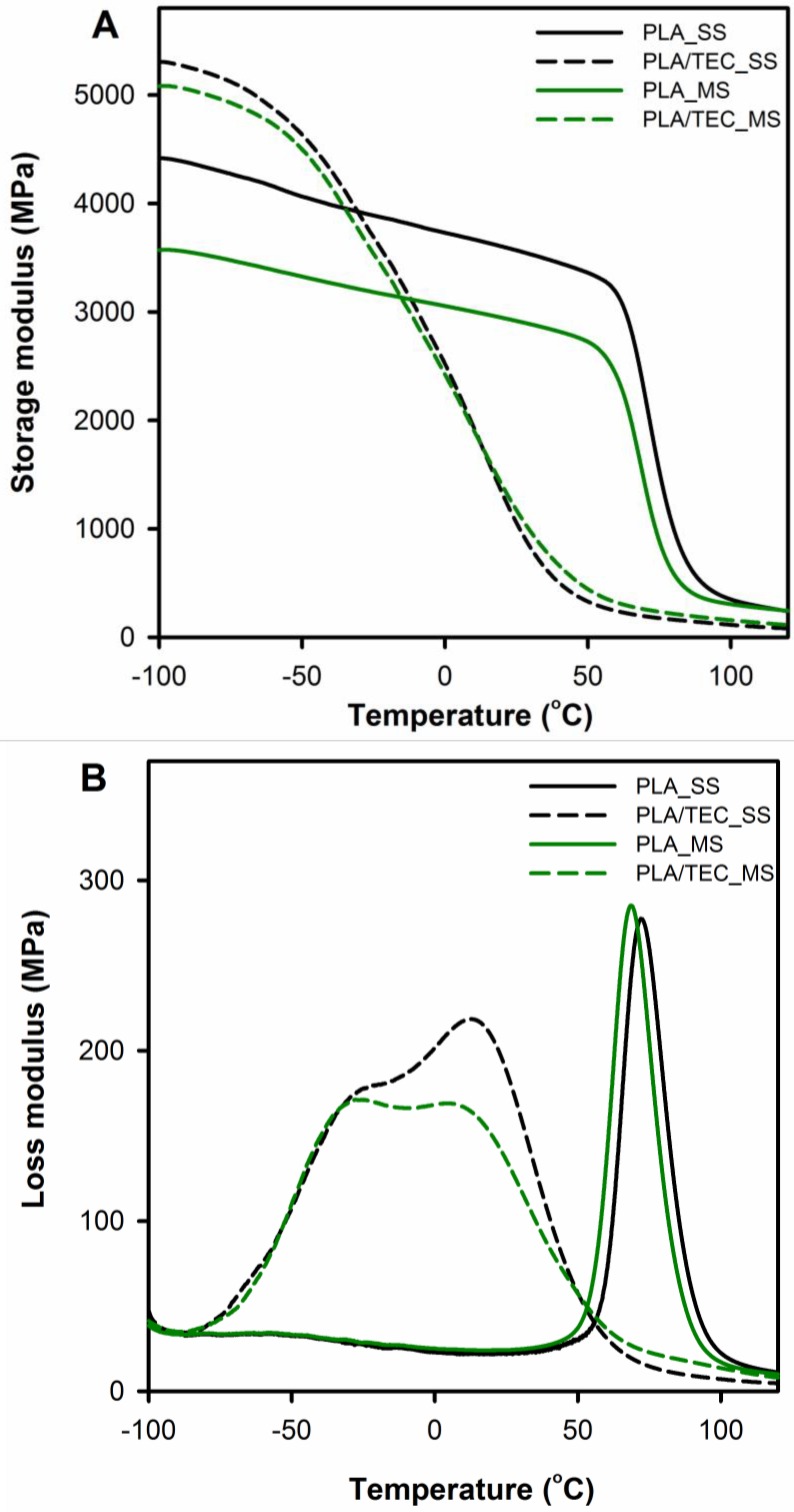
Temperature dependencies of storage modulus (**A**) and loss modulus (**B**) of PLA and PLA/TEC systems.

Figure 4 displays the storage (*E*′) and loss (*E*″) moduli curves comparing neat PLA with PLA/TEC systems samples. The relaxation temperature that can be associated with the glass transition was taken as *E*″ peak temperature (*T*_g_ (*E*″)) and is shown in Table 3 with results from DSC measurements.

In the DMTA curves of neat PLA samples (PLA_SS and PLA_MS), within the temperature range from −100 to 50 °C, the storage modulus *E*′ can be observed to fall by about 1000 MPa compared to the initial values. The decline indicates the molecular motions of the polylactide chains, which increase with increasing temperature to the rubbery region (significant decrease in storage modulus above 60 °C) and indicate α relaxation. The *E*′ curves for the plasticized PLA display different character of changes compared to the neat PLA, the *E*′ modulus of PLA/TEC systems samples starts to drop sharply at a temperature of about −60 °C. The observed shift of α relaxation for PLA/TEC systems toward lower temperatures is associated with the plasticization of PLA by the low molecular weight compound, TEC [38].

It is also worth noting that the samples of plasticized PLA in the temperature range below glass transition have a higher values of the storage modulus *E*′ compared to neat PLA. This increase in the storage modulus probably results from the “stiffening effect” of the polylactide matrix because of the introduced molecules of triethyl citrate. A similar phenomenon was observed by us for nanodecane/wax-modified polypropylene [21]. In the higher temperature range, i.e., above the glass transition of polylactide, the *E*′ modules of the PLA/TEC systems achieve lower values compared to pure polylactide. This means that as a result of incorporating the plasticizer into the polylactide matrix, the segmental movements of the polymer chains are facilitated.

Figure 4B displays temperature dependencies of loss modulus for neat and plasticized PLA. The *E*″ curves of the neat PLA exhibit single narrow peaks at 69 and 72 °C for PLA_MS and PLA_SS respectively, reflecting glass transition process in the amorphous phase. The *E*″ plots of the plasticized PLA show broad transition with double peaks, which are shifted toward lower temperatures compared to neat PLA samples. The broadening of the width of the *E*″ peak area associated with the energy required to activate the molecular mobility in the material is due to the presence of a plasticizer in the samples. This suggests that molecular mobility in plasticized PLA is more easily activated than in neat PLA [12]. The above phenomenon, together with the glass transition observed for the plasticized samples at a lower temperature than for the initial samples, indicates an efficiently carried out plasticization process of polylactide by introducing triethyl citrate into the polymer matrix at various stages of forming the final crystalline structure (SS and MS modification). The results are consistent with the results obtained by Pluta et al. and Zubrowska et al. for crystalline blends of polylactide with block copolymers of ethylene glycol and propylene glycol or with ethylene glycol derivative of POSS [30,39].

**Figure 5:**
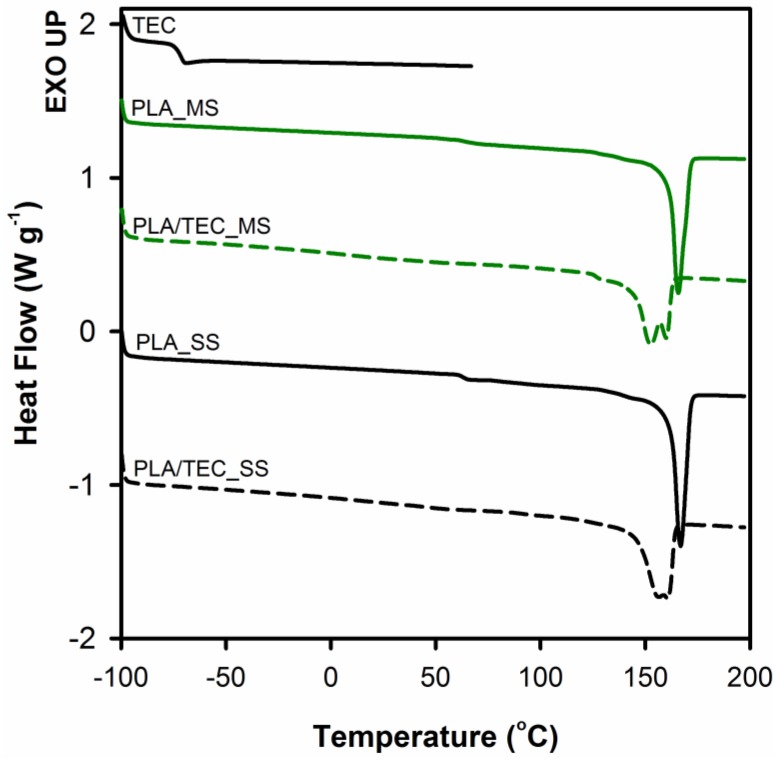
Differential scanning calorimetry (DSC) thermograms of polylactide and polylactide/triethyl citrate systems. Thermograms have been shifted along the vertical axis for better visualization.

Thermal behavior of neat PLA and PLA plasticized with TEC as well as the effect of the method of introducing triethyl citrate (by infusing to solidified polymer matrix or by blending before the solidification) on thermal properties of polylactide was analyzed by DSC. Figure 5 shows the endothermic peaks from the first heating curves of PLA and PLA/TEC samples. The thermal characteristics from DSC curves are summarized in Table 3.

From the Figure 5 it can be observed that samples of neat PLA display two thermal transitions: the glass transition temperature (*T*_g_) and the melting temperature (*T*_m_). The endothermic jump at temperatures around 63 °C is due to the glass transition, while endothermal peak within the temperature range from 120 to 180 °C is related to the melting temperature of crystals. The glass transition in the case of PLA/TEC samples becomes practically indistinguishable. The plasticizer of low molecular weight amorphous phase introduced into the polylactide causes broadening of this transition. Furthermore, the addition of TEC to PLA lowers the melting temperature of polylactide crystals both for PLA/TEC systems samples prepared by infusing to solidified polymer matrix and by blending before the solidification. The double melting peaks visible on the curves of plasticized samples PLA will be discussed later. The shift of *T*_m_ toward lower values of temperature is attributed to the plasticizing effect of the modifier, that is TEC. Our previous works [23,37] revealed that the change of the melting temperature of polypropylene/modifier systems may be caused by the decrease of equilibrated melting temperature of polymer crystal, *T*_m_, of an infinite stack of extended chain crystals, large in directions perpendicular to the chain axis and where the chain ends have established an equilibrium state of pairing. Additionally, we correlated this phenomenon with the changes (decrease) in the value of yield stress. In this work, we also noticed a reduction in yield stress for PLA plasticized with TEC samples compared to neat samples (see Figure 3).

The Figure 5 includes a curve of the heat flow as a function of temperature for citrate triethyl (TEC). As we can see no evidence of signals of triethyl citrate (*T*_g_ at about −73°C) was found on thermograms of PLA/TEC systems, independently of the method of introducing the modifier into the polylactide matrix. This suggests that the phase separation does not occur and the molecules of TEC are dispersed in the PLA matrix at the molecular level.

In summary, plasticization, independent of the way of introducing the plasticizer into polylactide matrix (by infusing to solidified polymer matrix or by blending before the solidification), leads to systems characterized by similar stability, structure (lack of phase separation process), and analogous mechanical and thermal properties. The observed differences in mechanical properties between MS and SS systems may result from the difference in the degree of crystallinity/crystal thickness of the PLA matrix itself, but not due to the difference in the effectiveness of the plasticization process. Therefore, we can conclude that the modification in the solid state can be an alternative for conventional method of plasticization of PLA-blending in a molten state. We are aware that the stage of introducing the plasticizer into the polymer matrix is time consuming, but it allows the use of compounds that are volatile or thermally unstable at the typical polylactide processing temperature (180 °C).

As previously mentioned, the DSC curves for that PLA/TEC systems samples show double melting peaks (see Figure 5). In the literature [8,40,41,42,43,44], these findings are most often correlated with the partial melting/recrystallization of metastable crystals, or the melting of a double population of crystals having different lamellar thickness, or the melting and reorganization of conformationally disordered crystals (α′-form crystal) into stable α-form crystals with two antiparallel aligned helical chain segments packed in an orthorhombic unit cell. However, the above models proposed to explain the occurrence of double melting peaks seem difficult to accept, especially for the PLA/TEC_SS system. In this system, before the introduction of the modifier, the polymer matrix was fully crystallized at a temperature (120 °C) significantly exceeding the temperature at which the modification process (70 °C) was carried out. Therefore, it is difficult to expect that the process of introducing the plasticizer will lead to a significant change of the crystalline structure of PLA matrix (according to one of the models presented above), which would result in the appearance of a double melting peak on DSC thermograms. Moreover, there are no visible transitions on the DSC thermograms that would indicate partial melting/recrystallization of metastable crystals or the melting and reorganization of conformationally disordered α′ crystals into stable α crystals.

**Figure 6:**
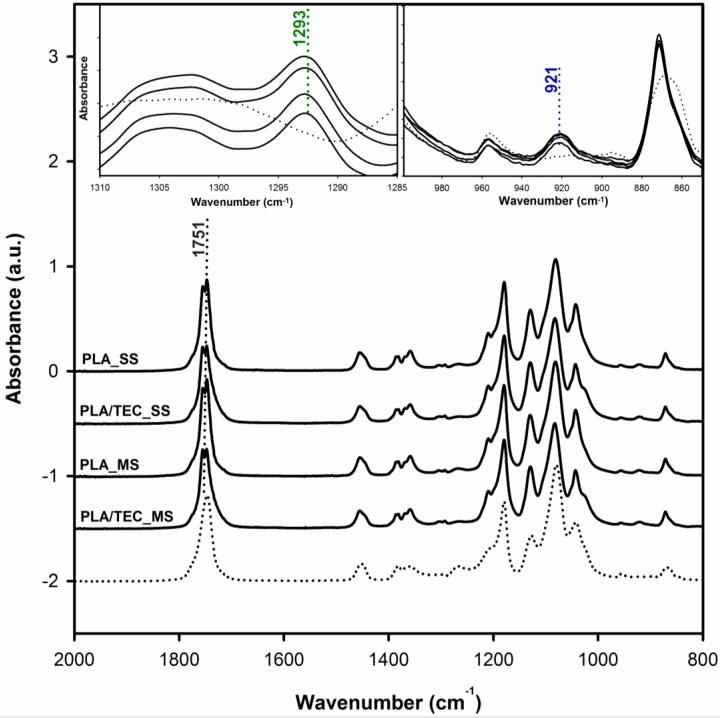
Fourier transform infrared (FTIR) spectra of neat PLA and PLA/TEC systems. The dotted line is the spectrum of an amorphous PLA sample.

Additional measurements using FTIR and X-ray techniques were performed to determine the location of the modifier and verify its impact on the crystalline structure of polylactide. FTIR spectroscopy is sensitive to the chain conformation or rotational conformers in the crystalline or disordered regions of polymers. Figure 6 shows FTIR spectra in the 2000–800 cm^−1^ regions of neat and plasticized polylactide samples. For comparison, the spectrum of an amorphous PLA is also given in Figure 6.

**Figure 7:**
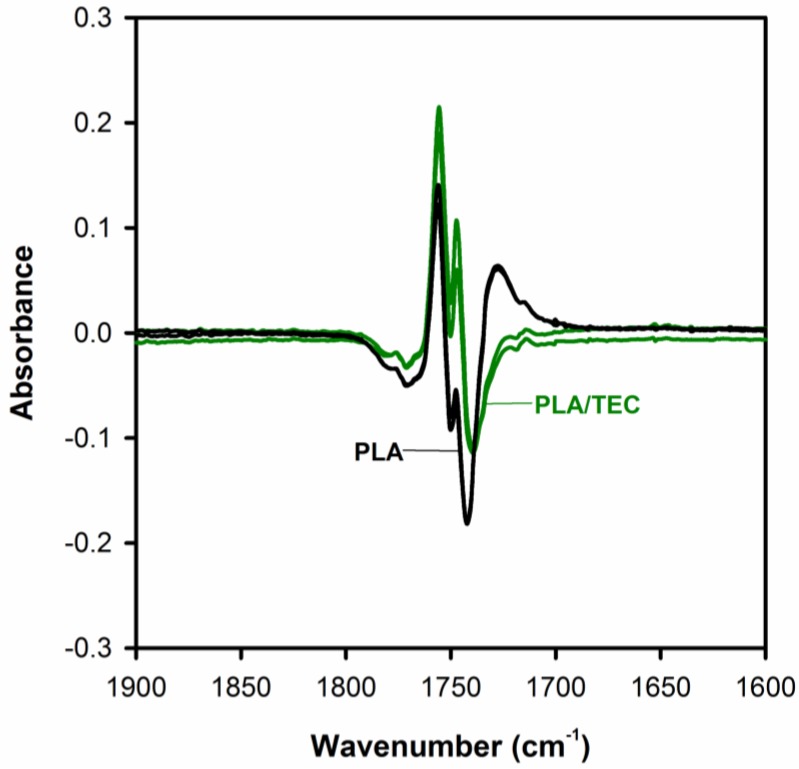
The difference spectra of PLA and PLA/TEC samples obtained from the FTIR spectra shown in Figure 6 by subtracting the amorphous PLA spectrum.

From the obtained spectral patterns it can be seen that all of the PLA samples exhibit the sharp bands around 921 and 1293 cm^−1^ (the inset of Figure 6), which are assigned to the coupling of C–C backbone stretching with the CH_3_ rocking mode and sensitive to the 10_3_ helix chain conformation of α crystals [45,46,47]. This finding indicates that both samples of neat and plasticized PLA consist of α crystals. From the absorption bands recorded on the spectra, two other areas are also sensitive to the structural changes of PLA. One of them is the C=O stretching vibration region of 1800–1700 cm^−1^, and the other is the region of 1500–1000 cm^−1^ which is involved in the CH_3_, CH bending, and C–O–C stretching vibration. Through the detailed analysis of the C=O stretching bands analysis (by use the difference spectra), we can verify whether the plasticization using triethyl citrate only applies to the amorphous phase of the semicrystalline polylactide, or whether it can somehow affect its crystalline phase. Figure 7 shows the difference spectra, calculated by subtracting the initial spectrum (amorphous PLA) from the rest of spectra, with three visible peaks in the *v*(C=O) spectral region.

The peaks at about 1756 and 1747 cm^−1^ correspond to the crystalline phase and a peak at about 1740 cm^−1^ to the amorphous phase of PLA. As shown in Figure 7, the band at 1743 cm^−1^ *v*(C=O) of PLA/TEC systems samples compared to the peak of neat PLA (band at 1740 cm^−1^) are shifted to higher wavenumbers, while the position of the bands at 1756 and 1747 cm^−1^ does not change. This observation is associated with the interactions between PLA matrix and triethyl citrate dispersed only in its amorphous phase.

**Figure 8:**
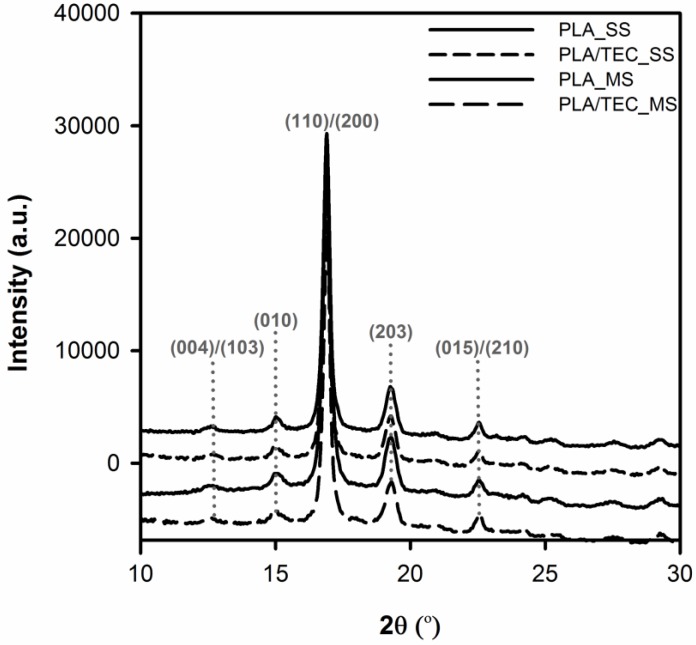
The diffraction patterns of neat (PLA_SS, PLA_MS) and plasticized PLA (PLA/TEC_SS, PLA/TEC_MS) samples.

The X-ray diffraction data support the above interpretation explicitly. From Figure 8 it is easy to notice that wide angle X-ray scattering (WAXS) profiles of neat and plasticized PLA samples are very similar—almost the same. The all PLA samples exhibit diffraction peaks at 2θ values of 12.61, 15.01, 16.89, 19.26, and 22.53°, which correspond to (004)/(103), (010), (110)/(200), (203), and (015)/(210) planes, respectively. The above reflections have the identical diffraction peaks with *α* form of PLA as reported [44,48,49,50]. Moreover, small diffraction peaks at 2θ = 20.86, 24.21, and 25.22° that correspond to reflections of (204), (115), and (206) planes of *α* form can be distinguished on WAXS profiles [51,52]. These findings undoubtedly confirms that cold crystallized PLA has no polymorphic crystalline transition. Therefore the possibility of interaction between the plasticizer (triethyl citrate) and the crystal structure of polylactide can be ruled out.

The small angle X-ray scattering (SAXS) results provide further evidence that the plasticizer introduced into the polylactide matrix penetrates it and accumulates in the amorphous phase of the polymer (1D SAXS patterns of PLA samples are shown in Figure S4 of Supplementary Material). Table 4 shows the values of long period and thickness of crystals determined from the SAXS measurements for neat PLA and PLA plasticized with TEC after matrix solidification. In addition, the table shows results for the PLA/TEC_SS system from which triethyl citrate was removed by methanol extraction (PLA/TEC_SSe).

**Figure 9:**
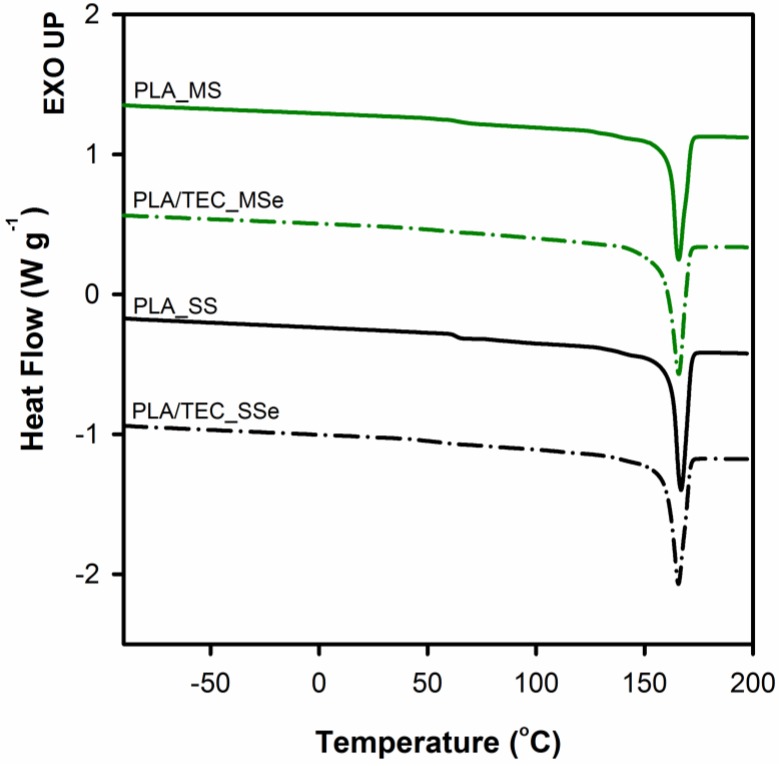
DSC thermograms of neat PLA (PLA_MS and PLA_SS) and PLA/TEC systems after plasticizer removal (PLA/TEC_MSe and PLA/TEC_SSe). Thermograms have been shifted along the vertical axis for better visualization.

As can be seen, all analyzed PLA samples have similar values of the parameters of crystals, which clearly indicates that both the process of introducing the plasticizer and its extraction do not change the thickness of the crystals. The change in the value of a long period of plasticized PLA (from 18.8 to 21.9 nm) is associated with an increase of interlamellar distance because of the swelling of the amorphous phase regions. Removal of previously introduced plasticizer as a result of extraction process returns the long period value to that of neat PLA (Table 4). The maximum of melting peak after removing the molecules of triethyl citrate (PLA/TEC_SSe and PLA/TEC_MSe samples) also shifts back to the values characteristic for the neat PLA (Figure 9). At the same time, the character of the melting peak of plasticized samples changes, namely after removal of the modifier a single melting peak analogous to the reference material can be observed on thermograms. This finding indicates that the process of introduction of the plasticizer into PLA matrix, irrespective of the method of its incorporation, does not change the crystalline structure of PLA and is a fully reversible process.

**Figure 10:**
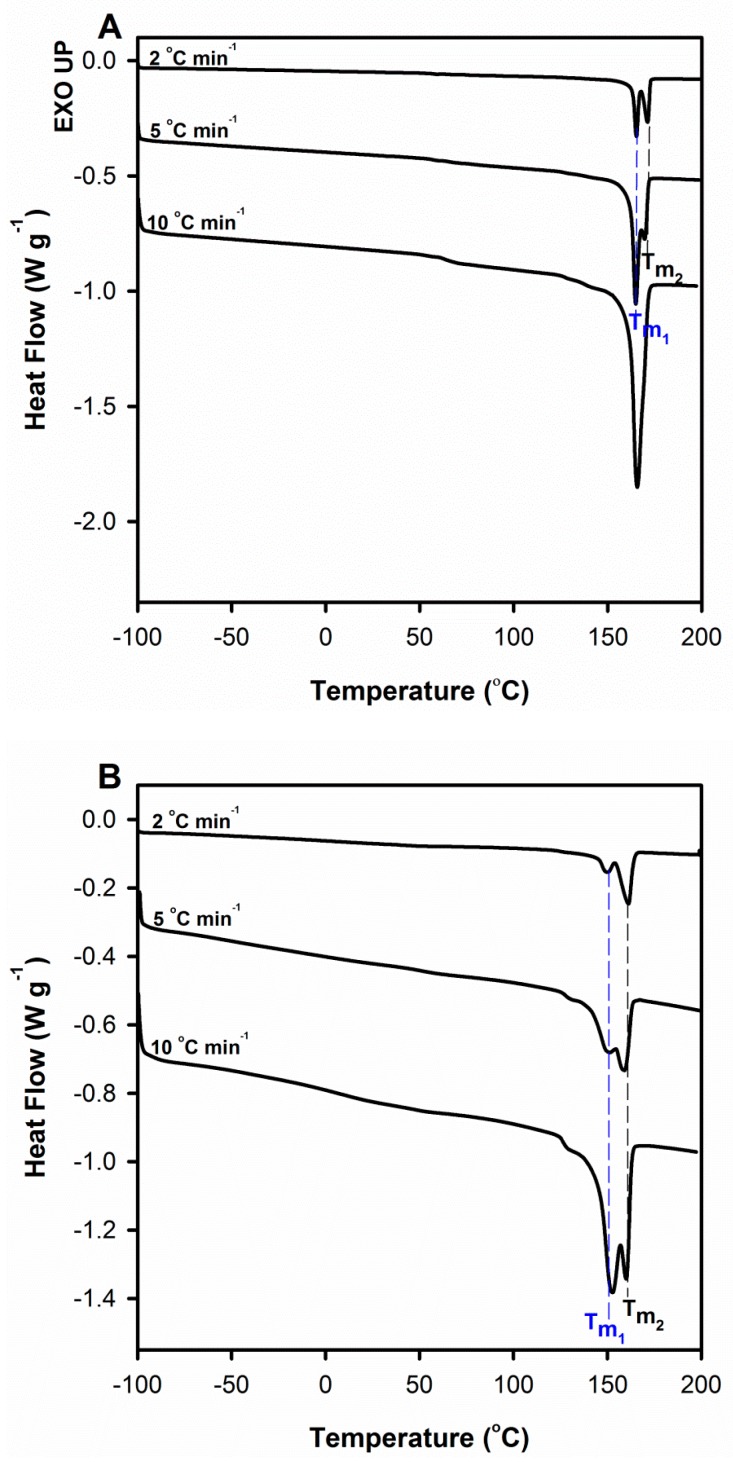
DSC melting thermograms of neat PLA (**A**) and PLA plasticized with TEC (**B**) at different scan rates. Thermograms have been shifted along the vertical axis for better visualization.

The multiple melting behavior observed on DSC thermograms of semicrystalline polymers may be affected by crystallization temperature, molecular weight, and also heating rate [41,43,53]. One of the proposed explanations for this phenomenon is the irreversible process of melting metastable crystals that may originate from the existence of metastable crystal forms as well as thin lamellae and defects in the crystals. It was noted that upon slow heating, rearrangement of the polymer molecules in crystals occurs with a higher degree of perfection or thicker lamellae, both depending on the heating rate. This approach seems most reasonable to elucidate the multiple melting characteristics of PLA/TEC systems. To verify this hypothesis, DSC thermograms for PLA and PLA/TEC systems at different heating rates (2, 5, and 10 °C min^−1^) were recorded (Figure 10).

It can be observed that in the case of plasticized samples double melting peaks appear at all heating rates (Figure 10A), whereas for pure samples only for lower scan rates, i.e., 2 and 5 °C min^−1^ (Figure 10B). At the same time, this additional peak is clearly located at higher temperatures, which means that the analyzed phenomenon is not kinetically induced. Moreover, with the decrease in the scan rates the melting peak at higher temperature (*T*_m2_) grew at the expense of the melting peak at lower temperature (*T*_m1_) while the total endothermic area remained unchanged. The above phenomena are associated with the process of improvement of crystal perfection/thickness that was possible under the conditions of low heating rates. Plasticization of polymers improves the chain mobility, and therefore the rearrangement of polymer molecules upon heating to crystals with a higher degree of perfection for PLA/TEC samples will require less time than for neat PLA.

To better clarify the process of improvement of crystal perfection DSC measurements for neat PLA after annealing at T = 155 °C for 10 min were performed.

**Figure 11:**
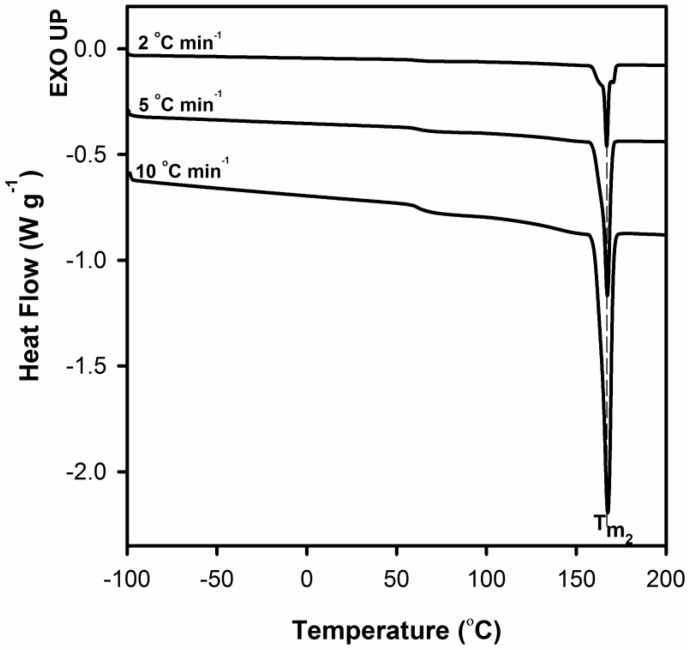
DSC melting thermograms of neat PLA after annealing at 155 °C for 10 min at different scan rates. Thermograms have been shifted along the vertical axis for better visualization.

On the DSC curves in the Figure 11, regardless of the heating rate, only the melting peak *T*_m2_ is clearly visible. This means that the molecular rearrangement took place during the annealing, as a result of which the crystal structure of the polymer became more perfect/regular. WAXS profiles collected for PLA samples before and after annealing at 155 °C for 10 min served to determine the crystallites length (length of undisturbed fragment of crystal) in normal direction to (200)/(110) and (203) crystallographic planes with the use of Scherrer’s Equation (1). In order to evaluate the half-width of selected peaks the deconvolution process of WAXS profiles with the use of WAXSFit software was performed. During deconvolution process the presence of both crystalline (α-form) and amorphous component was taken into account. The crystallite length was determined in the normal direction to the (200)/(110) planes after annealing increases from 35.7 to 43.6 nm, while for the (203) plane from 20.8 to 24.0 nm. This finding clearly indicates the process of improvement of crystal perfection.

## 4. Conclusions

Polylactide because of its inherent brittle behavior was plasticized with triethyl citrate—low molecular weight compound. The plasticization process was performed in two ways: by infusing the plasticizer into the amorphous phase regions of solidified polymer and by melt blending the polylactide/triethyl citrate components before the solidification. Analysis of the plasticizer desorption kinetics curves showed that the PLA/TEC systems are characterized by a good stability. Using the scanning electron microscopy, no heterogeneity was found, which proves the formation of a phase enriched in a plasticizer instead of distinct inclusions of the plasticizer. Thermal analysis (DMTA and DSC measurements) demonstrated that the introduction of a triethyl citrate into the PLA matrix leads to a reduction in both the glass transition temperature and the melting temperature of the crystals. The shift of temperature transitions toward lower values results from plasticization and is observed for samples obtained by the modification in the solid and molten state. Moreover, thermal analysis revealed broadening of the glass transition in the PLA/TEC systems compared to reference samples of PLA. The incorporation of the plasticizer into the polymer matrix, regardless of its solidification state, also resulted in the reduction of the yield point.

The plasticization processes, independent of the way of introducing the plasticizer into polylactide matrix (by infusing to solidified polymer matrix or by blending before the solidification), leads to systems characterized by similar stability, structure (lack of phase separation process), and analogous mechanical and thermal properties. Some differences in mechanical properties between MS and SS systems result primarily from the difference in the degree of crystallinity/crystal thickness of the PLA matrix itself; these differences are not the result of a difference in the effectiveness of the plasticization process. Based on the results of the research, it can be concluded that the plasticization process after solidification of polylactide is an alternative to the conventional method of modification-blending in a molten state. In addition, this new approach to plasticization process was utilized for correct interpretation of thermal properties of PLA/TEC systems. It turned out that double melting behavior does not result from the melting of a double population of crystals with different lamellar thickness, or the melting of both the α′ and α crystalline phase but is associated with the improvement of perfection of crystalline structure of polylactide during DSC heating process.

## Figures and Tables

**Table 1:** Content of triethyl citrate in the polylactide/triethyl citrate (PLA/TEC) systems (solid and molten state modification).

Sample	Triethyl Citrate Content		
	Weight Increment (wt.%)	TGA (wt.%)	Methanol Extraction (wt.%)
PLA/TEC_SS	14.1	-	-
PLA/TEC_MS	-	15.7	15.4

**Table 2:** The results of tests of mechanical properties of neat PLA and PLA/TEC systems.

Sample	Yield Stress *σ*_y_ (MPa)	Stress at Fracture *σ*_f_ (MPa)	Strain at Fracture *ɛ*_f_ (%)
PLA_SS	33.4	31.5	23.2
PLA/TEC_SS	16.0	11.8	216.2
PLA_MS	27.8	24.2	75.2
PLA/TEC_MS	12.1	10.2	488.5

**Table 3:** Thermal data obtained by DSC and dynamic mechanical thermal analysis (DMTA) measurements for PLA and PLA/TEC systems.

Sample	*T*_g_ (°C)	*T*_m_ (°C)	Δ*H*_m_ (J g^−1^)	*X*_C_ (%)	*T*_g_ (*E*″) (°C)
PLA_SS	63.5	166.8	47.7	33.4	72.2
PLA/TEC_SS	n.a.	156.5/160.4	48.2	33.7	−22.5/12.8
PLA_MS	62.8	165.8	44.7	31.3	68.7
PLA/TEC_MS	n.a.	154.2/160.2	45.1	31.5	−25.6/5.6

n.a.—not appliacable.

**Table 4:** Long period and thickness of lamellae of polylactide, polylactide/triethyl citrate system, and polylactide/triethyl citrate system after plasticizer removal determined by correlation function method.

Sample	Long Period (nm)	Thickness of Lamellae (nm)
PLA_SS	18.8	7.6
PLA/TEC_SS	21.9	7.8
PLA/TEC_SSe	18.6	7.7

## Supplementary Materials

The following are available online at https://www.mdpi.com/2073-4360/12/3/561/s1. Figure S1: Kinetics of triethyl citrate sorption by polylactide film. Figure S2: TGA thermograms in air for polylactide (PLA), triethyl citrate (TEC), and their blends. Figure S3: Kinetics of triethyl citrate desorption for PLA/TEC systems samples. Figure S4: 1D SAXS patterns of polylactide (PLA), polylactide/triethyl citrate system (PLA/TEC_SS), and polylactide/triethyl citrate system after plasticizer removal (PLA/TEC_SSe).
