# Supplementary material for: Plasticization of Polylactide after Solidification: An Effectiveness and Utilization for Correct Interpretation of Thermal Properties
Source: Polymers (Basel). 2020 Mar 4;12(3):561. doi: 10.3390/polym12030561 (PMC7182837; doi:10.3390/polym12030561)
Supplement: Supplementary file 1 [file polymers-12-00561-s001.pdf]

Supplementary data for Polymers

Plasticization of polylactide after solidification: an effectiveness and utilization for correct interpretation of thermal properties

Marta Safandowska*, Artur Rozanski*, Andrzej Galeski
Centre of Molecular and Macromolecular Studies, Polish Academy of Sciences, Sienkiewicza 112, 90-363 Lodz, Poland
* msafan@cbmm.lodz.pl; rozanski@cbmm.lodz.pl

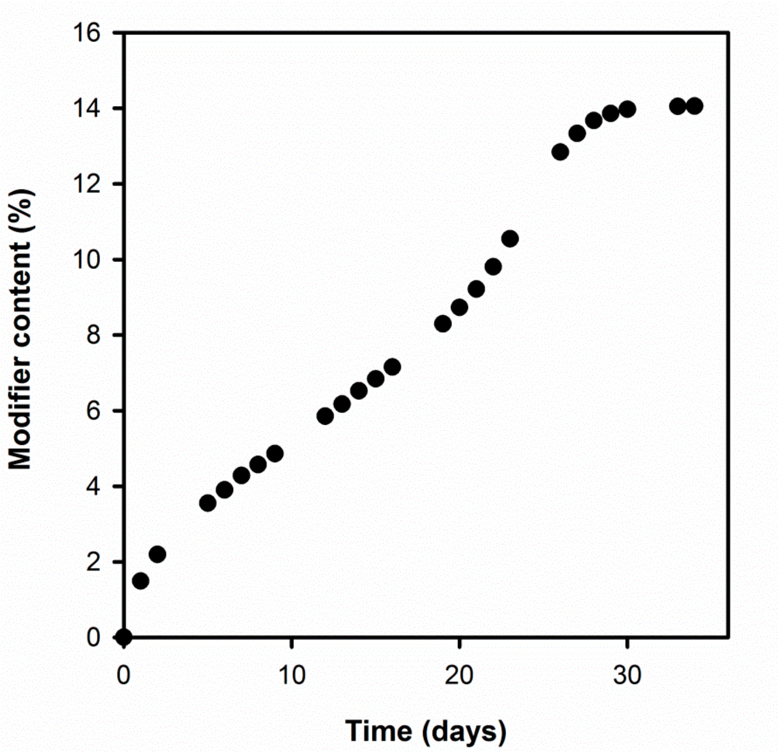

Figure S1. Kinetics of triethyl citrate sorption by polylactide film.

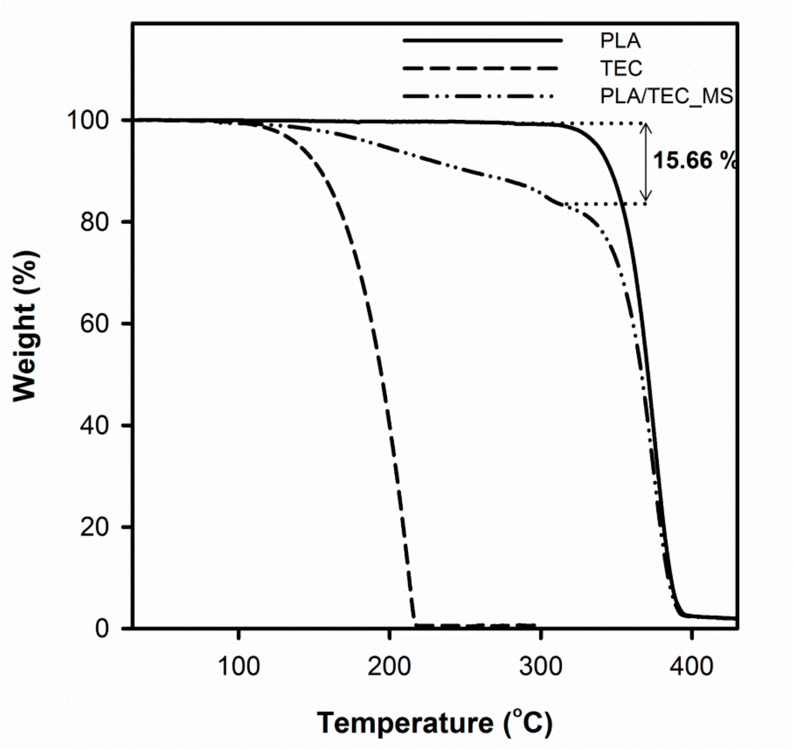

Figure S2. TGA thermograms in air for polylactide (PLA), triethyl citrate (TEC) and their blends.

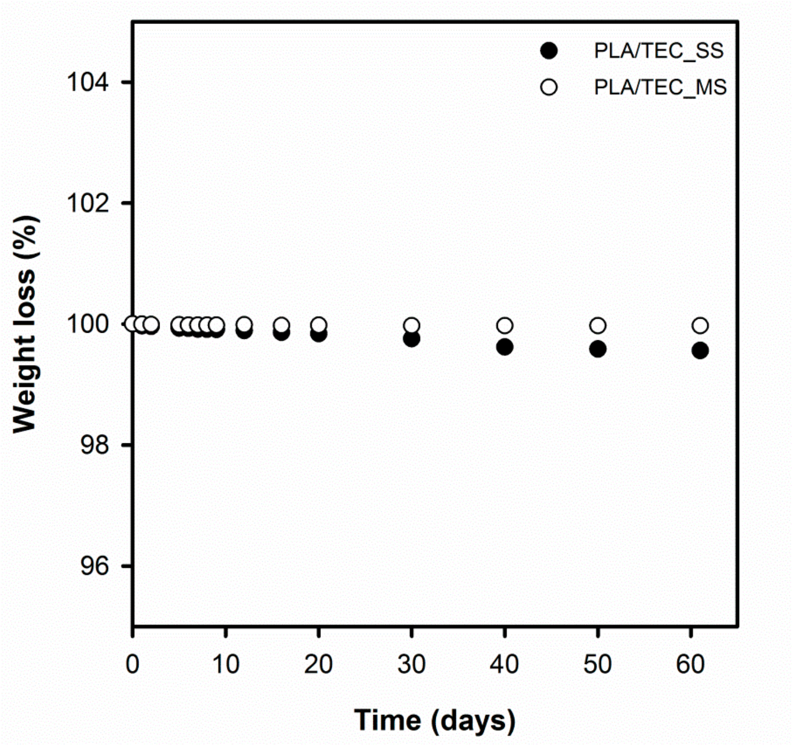

Figure S3. Kinetics of triethyl citrate desorption for PLA/TEC systems samples.

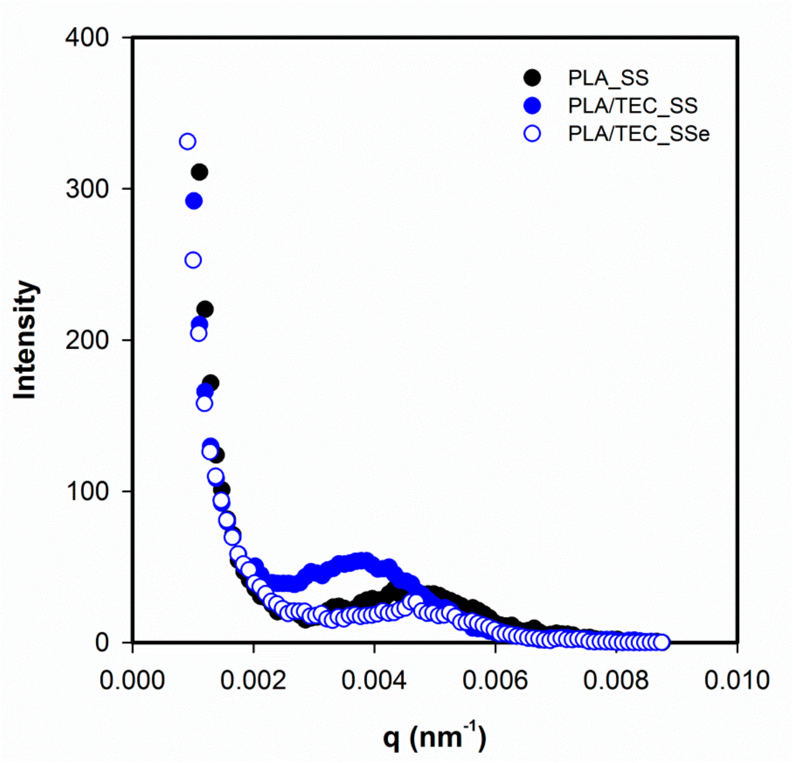

Figure S4. 1D SAXS patterns of polylactide (PLA), polylactide/triethyl citrate system (PLA/TEC_SS) and polylactide/triethyl citrate system after plasticizer removal (PLA/TEC_SSe). The samples was prepared by solid state modification method (SS).
